# Selections

**Published:** 1863-02

**Authors:** 


### SELECTIONS.

*From Braithwaite's Retrospect.*

*Subcutaneous Injection of Quinine.*—Dr. Chasseaud finds from a great number of experiments, that one or two grains of quinine in solution injected into the cellular tissue of the arm, are equally, if not more efficient in arresting fever than the large doses hitherto found necessary. This method is also free from the unpleasant symptoms sometimes produced when a large dose of quinine is taken by the mouth. Make a saturated solution of quinia in alcohol, and of this, inject as much as is equivalent to two grains, under the skin of the arm, avoiding the large veins. One curious effect produced, is that the patients generally fall into a quiet sleep for some hours. The great expense of sulphate of quinine, apart from the merits of the discovery itself, render this of great importance.—Dr. J. M'Craith.

*Typh Fevers.*—Typhus and typhoid fevers are but different types of the same disease, that is, arising from one common cause. No uniform plan can be adopted in treating fever, each single instance must be treated by itself, and symptoms must be met as they arise. Provided it be seen early the typhoid type is, of the many forms of fever, the one most amenable to treatment. When required by the severity of the local symptoms, never hesitate to have leeches applied over the right iliac region, and, in some cases, a blister over the same part is very useful, and is often followed by the best results. In the diarrhœa of typhoid fever the best remedy is the dilute sulphuric acid, in the proportion of one to three drachms to the eight-ounce mixture. Such medicines as chalk, gallic acid, lead and opium are often unavailingly used. The rule should be to gradually lessen the diarrhœa if too severe, never suddenly to stop it. In mild cases use the acid infusion of roses, and, when there is pain, from two to six drops of laudanum in each dose of the mixture, commonly answers well. When there are signs of irritation in the colon, and more especially when there is tenesmus, an anodyne enema acts like a charm. The use of salines or alkalies in any disease of a lowering type is to be avoided. Even carbonate of ammonia is too indiscriminately used. A very few doses of it will frequently bring on diarrhœa. Perhaps stimulants as a class are too indiscriminately used—brandy, wine, and beef-tea are constantly spoken of as being given to the same patient. Judgment must be exercised, for the effects are not the same. Thus wine may be given with much less risk than beef-tea.—Dr. H. Kennedy.

*Typhoid and Scarlet Fever—Chlorine and Milk.*—From the very commencement of a fever there is a very great loathing of all kinds of nourishment; the bare suggestion of food creates a shudder. Yet milk will always be found grateful. It is the most unirritating and digestible food possible. Dr. Thielman writes that "he had never seen delirium occur during the progress of any fever under the influeuce of a full diet of milk, as after broth." If the stomach will bear it gruel may be given, made as thick as good cream, and with two-thirds milk. After the first two or three days of antiphlogistic treatment, the internal and external use of chloroform and chlorine is most beneficial. Let the chloroform be given in small doses at first, and increased; after each dose a great calmness and inclination to sleep is produced, unattended with any adverse cerebral symptoms. The chlorine may be administered internally either as chlorate of potash or very dilute hydrochloric acid. Chlorine, thus given, at once destroys the putrid effluvia thrown off from the various secretory surfaces of the body, so injurious both to the patient and his attendants. The body may be sponged twice a-day with tepid chlorinated water. Change of linen and of bed, where two beds can be plaeed in the same room, will contribute greatly to the general welfare and convalescence of the patient. Wines, all soups, and jellies, should be deferred until the termination of the febrile symptoms.—C. T. Edwards.

*Chlorine and Chlorine Acids.—Nitric and Nitrous Acids.*—There is a remarkable difference between the action of the chlorine acids and nitric and nitrous acids in scarlatina. The former appears to exercise a direct action upon the morbid poison, while the latter possesses no therapeutical effects. The chlorine acids are to the morbid poison of scarlatina what the nitric and nitrous acids are to typhoid fever. These oxygen acids have proved the most valuable therapeutic agents in typhoid fever. These two sets of acids take precedence of ammonia as therapeutic agents. The mineral acids are, however, qúite contraindicated when cerebral symptoms are present. The acids must not be combined with other remedial agents which tend to mask their action, but a few drops of ipecacuanha wine and syrup of poppies may be added to nitric acid, or to the hydrochloric, should bronchial or pulmonary complications require; and where tonic treatment is necessary, they may be combined with bark and quinine. If nitric acid has been given in excess, the tongue becomes very red, and the use of the remedy must be desisted from. The rapidity with which nitric acid clears away the dusky hue, and brightens the countenance, by decomposing the morbid poison of the blood, is very remarkable in case of typhoid fever. This dusky hue does not depend upon feeble action of the heart, for ammonia is found often to increase the mischief, probably from its having an affinity for the morbid matter contained in the blood.—Dr. H. Osborn.

*Zymotic Diseases.*—Let that be admitted, of which there can be little question, that zymotic diseases depend on the presence and action of specific ferments in the blood, the question arises, is it possible to neutralize them? (Claude Bernard has established the fact that fermentation may arise in the blood and give rise to poisonous principles which may in their turn produce certain grave accidents in the living frame.) We possess in sulphurous acid, when combined with salifiable bases, a means of controlling and neutralizing morbid ferments in the blood of living animals, without in any way vitiating its qualities, so as to render it in any way incapable of maintaining life. Not only sulphurous acid, but also its combinations with earths and alkalies, such as the sulphites of soda, potash, magnesia, and lime, possess in a supreme degree the power of arresting all known organic fermentations, and putrefactive metamorphoses of animal solids and liquids, It never acts as a poison on the living organism as do many other substances well known for their antiseptic properties.—Dr. J. Polli.

*Delirium Tremens.*—The following is Dr. Laycock's rational plan of treating delirium tremens without the use of opiates. Let the patient be put to bed, his hands and face washed, and let the room be kept cool and sweet. No mechanical restraint must be attempted, but the patient governed by a calm, gentle, yet firm and positive manner. Food should be administered in a concentrated form, in small quantities, every two or three hours. If the breath smells of drink, await the elimination of the poison, unless there be reason to suspect an overdose, when a gentle emetic may be prescribed, and the stomach emptied. When food has not been taken for several days, and the hallucinations are of a frightful or distressing kind, and especially when the pulse is very quick and feeble, the first sound of the heart heard indistinctly, the tongue coated, œdematous, and flat or indented at the edges, wine and brandy may be administered medicinally with advantage.—Dr. T. Laycock.

A curious case is related in which delirium tremens did *not* result from drink, but from great mental excitement. Forty drops of laudanum with fifteen drops of chloroform, induced sleep within half an hour of administration, This combination is one of much value in the treatment of delirium tremens.—Mr. A. Murphy.

*Croup—Veratrum Viride.*—It is probable that veratrum viride will prove a substantial addition to our means of controlling inflammatory disease. It acts clearly as a depressor of the circulation. Given in croup it causes sickness, less frequent pulse, and general relief to the symptoms. Two minims of the tincture should be given every hour.—Dr. C. Handfield Jones.

*Pleurisy.*—Blisters at the commencement of pleurisy invariably protract the duration of the inflammation, and make it more severe. The property of cantharides is to cause and augment that very fibrinous state from which the membrane is already suffering. Exposure to cold and to changes of temperature by baths and the like, makes it worse, as do strained postures of the body and exercise. Opiates also cover up the evil with an anæsthetic mask, and prevent the patient knowing how he really is. Mercury, again, is an unnecessary call upon the whole system to make destructive sacrifices for the sake of a very small and not important member. Purgatives do no good, and expose the patient to catch cold at the water-closet. On the other hand, the application of six to twelve leeches gives immediate relief. After these come off, apply a very large poultice, and continue the use of poultices for several days. Take care not to follow the application of invigorating warmth by the depressing influence of cold. The poultice must be kept on hot until all pain is gone, and the breath can be drawn quite freely and easily.—Dr. T. K. Chambers.

---

Dr. H. B. Moore, of Coldwater, Michigan, reports in the *Chicago Medical Journal* for January, a case of strangulated inguinal hernia, in which three and a half inches of mortified intestine were removed by excision, which was accomplished by raising the intestine and dividing the mortified portion entire down to the mesentery. The opposing ends were brought together and secured by four stitches or interrupted sutures. On the fifth day after operation fæcal evacuation occurred, and again the eighth day freely. Upon examination of the bowel removed, it was found to contain, besides some fæcal matter, three desert spoonsfull of blackberry seeds, four plum stones, and several fragments of chicken-bone from one-fourth to five-eighths of an inch in length. Patient said she masticated her food imperfectly on account of defective teeth. Two months after operation, patient considered herself well, the bowels having resumed their natural action.

